# Iron-induced calcification in human aortic vascular smooth muscle cells through interleukin-24 (IL-24), with/without TNF-alpha

**DOI:** 10.1038/s41598-017-19092-1

**Published:** 2018-01-12

**Authors:** Sayuri Kawada, Yasuyuki Nagasawa, Mutsuki Kawabe, Hideki Ohyama, Aritoshi Kida, Nahoko Kato-Kogoe, Masayoshi Nanami, Yukiko Hasuike, Takahiro Kuragano, Hiromitsu Kishimoto, Keiji Nakasho, Takeshi Nakanishi

**Affiliations:** 1 Department of Internal Medicine, Division of Kidney and Dialysis, Hyogo College of Medicine, 1-1 Mukogawa-Cho, Nishinomiya, Hyogo, Japan; 2 Department of Pathology, Hyogo College of Medicine, 1-1 Mukogawa-Cho, Nishinomiya, Hyogo, Japan; 3 Department of Oral and Maxillofacial Surgery, Hyogo College of Medicine, 1-1 Mukogawa-Cho, Nishinomiya, Hyogo, Japan

## Abstract

In CKD patients, arteriosclerotic lesions, including calcification, can occur in vascular smooth muscle cells in a process called Moenckeberg’s medial arteriosclerosis. Iron overload induces several complications, including the acceleration of arteriosclerosis. However, the relationship between Moenckeberg’s arteriosclerosis in vascular smooth muscle cells and iron accumulation has remained unknown. We tested the accelerated effect of iron on calcification in cultured human aortic vascular smooth muscle cells (HASMCs). After establishment of this model, we performed a microarray analysis using mRNA from early stage culture HASMCs after iron stimulation with or without TNF-alpha stimulation. The role of interleukin-24 (IL-24) was confirmed from candidate genes that might contribute to calcification. HASMCs demonstrated calcification induced by iron and TNF-alpha. Calcification of HASMCs was synergistically enhanced by stimulation with both iron and TNF-alpha. In the early phase of calcification, microarray analysis revealed up-regulation of IL-24. Stimulation of HASMCs by IL-24 instead of iron induced calcification. The anti-IL-24 antibody reversed the effect of IL-24, supporting the important role of IL-24 in HASMCs calcification. In conclusion, iron-induced calcification in vascular smooth muscle cells occurred via IL-24, IL-24 was increased during the calcification process induced by iron, and IL-24 itself caused calcification in the absence of iron.

## Introduction

The concept of chronic kidney disease (CKD) was established because of a high incidence of cardiovascular events; therefore, arteriosclerosis diseases have occupied an important part of CKD^1,2^. In CKD patients, arteriosclerotic lesions, including calcification, can occur in vascular smooth muscle cells in a process called Moenckeberg’s medial arteriosclerosis^2,3^.

Supplementation with iron is commonly used as an adjunctive therapy for anemia because CKD patients usually suffer from renal anemia. Iron is an important renal anemia treatment, in combination with erythropoiesis-stimulating agents^4–8^. However, iron overload has been considered to have some relationship with several complications, including acceleration of arteriosclerosis^9,10^. Iron accumulation has been observed in human atherosclerotic plaque lesions^11^. Our group reported that tumor necrosis factor-alpha (TNF-alpha) induced iron sequestration and oxidative stress in human endothelial cells^12^. In terms of Moenckeberg’s arteriosclerosis in vascular smooth muscle cells, the mechanism is believed to be similar to the mechanism of vascular calcification^13–15^. Hyperphosphatemia in uremic conditions enhances calcification, resulting in worsening of mortality in CKD patients^15,16^. Our hypothesis is that iron accumulation under uremic conditions with hyperphosphatemia might be related to calcification of vascular smooth muscle cells (Moenckeberg’s arteriosclerosis). However, the relationship between Moenckeberg’s arteriosclerosis in vascular smooth muscle cells and iron accumulation remains unknown.

In this study, we tested the accelerated effect of iron on calcification in cultured vascular smooth muscle cells. After establishment of this model, we performed a microarray analysis using mRNA from early stage culture vascular media cells after iron stimulation with or without TNF-alpha stimulation. The role of interleukin-24 (IL-24) was confirmed from the candidate genes that might contribute to calcification in vascular media cells.

## Results

### The effects of iron and TNF-alpha stimulation on calcification

**Figure 1:**
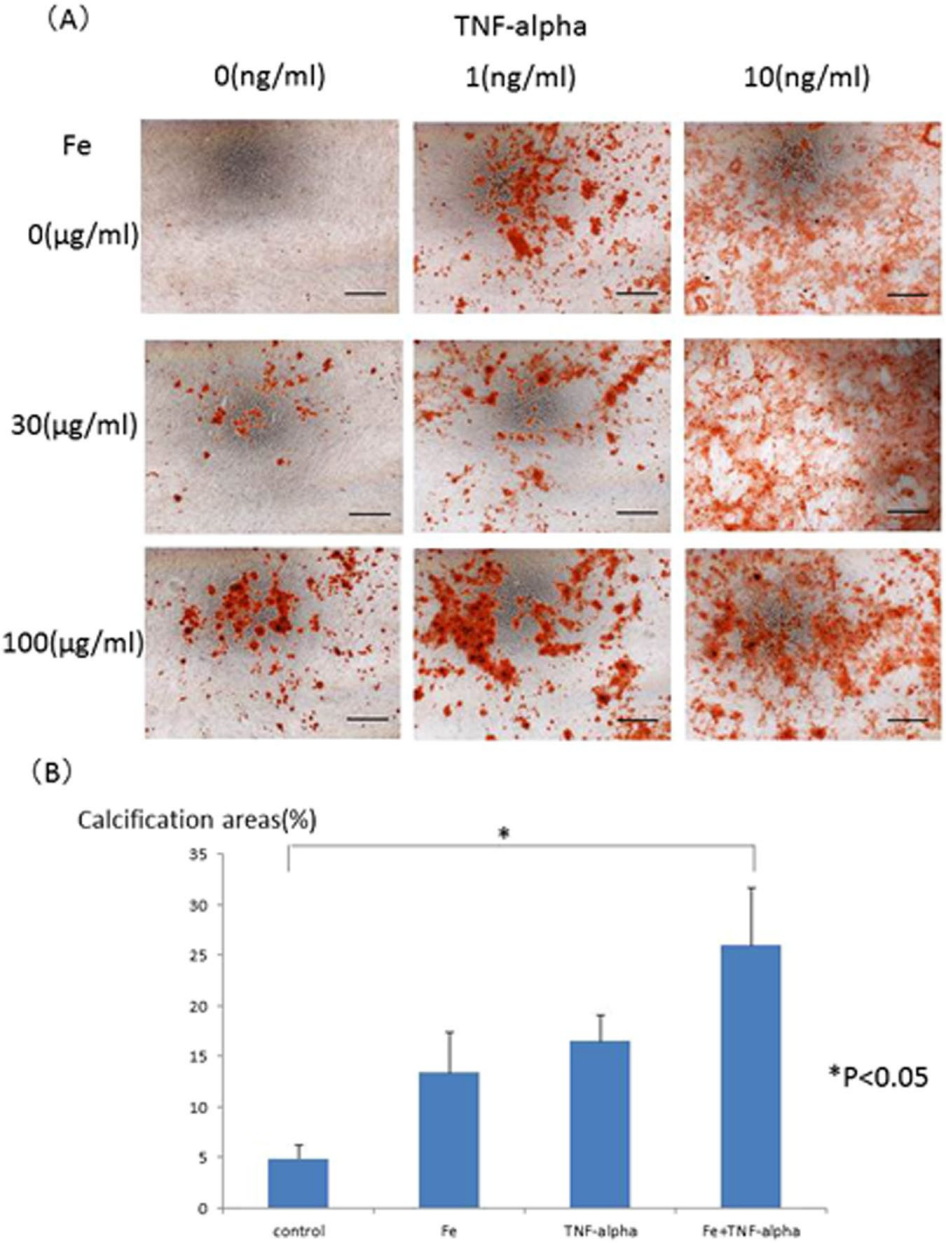
(**A**) Typical images of calcification of human aorta vascular smooth muscle cells induced by iron, TNF-alpha or both iron and TNF-alpha. To induce human aortic smooth muscle cells (HASMCs) calcification, the cells were incubated with the calcification medium for 15–21 days, supplemented with holo-transferrin (holo-Tf) (0, 30, or 100 µg/mL) and TNF-alpha (0, 1, or 10 ng/mL). Mineralized cell nodules were stained with Alizarin red, and typical calcification images in HASMCs are shown. Iron and TNF-alpha stimulation enhanced calcification. Black bars indicate 500 micrometers. (**B**) Quantification of calcification on HASMCs induced by iron, TNF-alpha or both iron and TNF-alpha by ImageJ software. The calcification areas of HASMCs stained with Alizarin red were quantified by ImageJ software. Iron induced HASMCs calcification, and TNF-alpha induced HASMCs calcification in a dose-dependent manner. Both 100 µg/mL iron (holo-transferrin) and 1 ng/mL TNF-alpha synergistically induced HASMCs calcification. These experiments used two cell lines of HASMCs.

**Figure 2:**
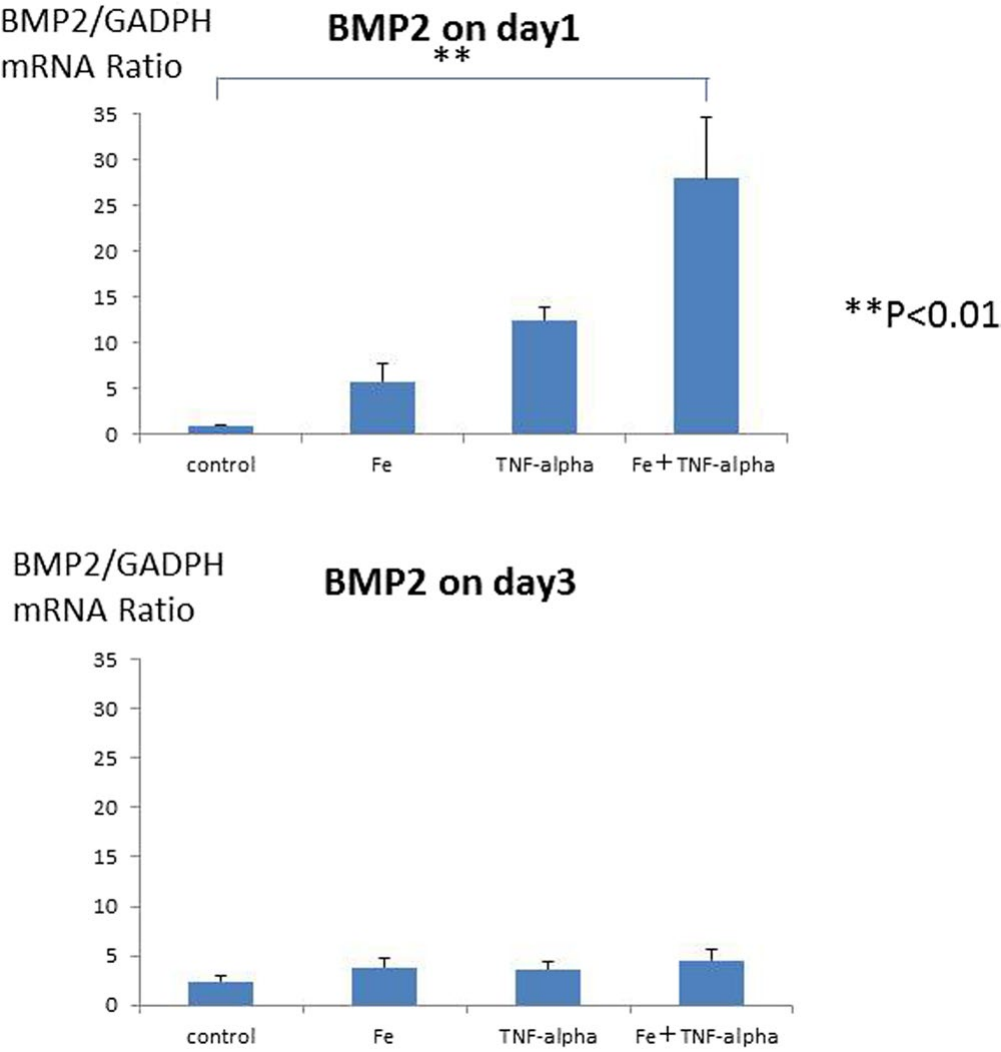
BMP2 expression was evaluated by real-time quantitative PCR induced by iron, TNF-alpha or both iron and TNF-alpha. To confirm the calcification pathway, BMP2 expression was evaluated by real-time quantitative PCR. BMP2 expression was elevated by 100 µg/mL iron (holo-transferrin) or 1 ng/mL TNF-alpha on day 1, but elevation of BMP2 expression was not observed on day 3. These experiments used one cell lines of HASMCs.

HASMCs were incubated with the calcification medium for 15–21 days with iron and/or TNF-alpha stimulation. Mineralized cell nodules were stained using Alizarin red. Iron and TNF-alpha stimulation enhanced the calcification of HASMCs in phosphate-containing calcification medium. Typical calcification images of HASMCs are shown in Fig. 1A. The calcification areas were quantified by ImageJ software, and the quantification results are shown in Fig. 1B. Iron (more than 100 µg/mL holo-transferrin) induced HASMC calcification. TNF-alpha (more than 1 ng/mL) also induced HASMC calcification in a dose-dependent manner up to 10 ng/ml. Interestingly, 100 µg/mL iron and 1 ng/mL TNF-alpha synergistically induced HASMC calcification. To confirm the calcification pathway, To confirm the safety of iron on human aortic smooth muscle cells (HASMCs), the cells were cultured with the calcification medium for 15–21 days supplemented with holo-Transferrin (holo-Tf) (0, 100, 1000 or 10000 µg/mL) and TNF-alpha (0, 1, or 10 ng/mL). Mineralized cell nodules were stained with Alizarin red, and typical calcification images in HASMCs are shown (Supplemental Fig. 1). The high concentration (10000 µg/mL) stimulation induced cell death and suppressed calcification. BMP2 expression was evaluated by real-time quantitative PCR. BMP2 mRNA levels were elevated by iron and/or TNF-alpha stimulation on day 1, but the BMP2 expression level returned to baseline under all conditions on day 3 (Fig. 2). The time course of BMP2 mRNA levels was also evaluated, and the BMP2 mRNA returned to the basal level from day 3 until the end of the calcification period (Supplemental Fig. 2).

To make the calcification process clearer, several calcification markers were evaluated with regard to mRNA levels. Runx2 and MSX2 were elevated on day 6 in response to calcification medium stimulation (Supplemental Figs 3 and 4). The RANKL level was significantly elevated by TNF-alpha and iron stimulation on day 9 and day 12, respectively (Supplemental Fig. 5). The hOPG level seemed to increase without significance (Supplemental Fig. 6). ALP activity seemed to increase on day 3 without significance (Supplemental Fig. 7).

We have no information of ascorbic acid in this calcification medium. At least, no ascorbic acid was added in our experimental protocol. Therefore, no ascorbic acid might be present in the medium, so it is possible that crystals were not deposited into collagen fibrils.

### Microarray analysis of the early response to iron stimulation

Microarray analysis was performed using the RNA from HASMCs on day 1 with or without iron or TNF-alpha stimulation to reveal the early genetic mechanism of calcification enhancement in HASMCs induced by iron or TNF-alpha stimulation, compared to control HASMCs. Genes in HASMCs with both iron and TNF-alpha stimulation that were increased more than ten-fold compared to the controls are shown in Table 1, including the increased levels between the controls and HASMCs with iron stimulation and between the controls and HASMCs with TNF-alpha stimulation. Among these genes, IL-24 was synergistically increased by both iron and TNF-alpha stimulation, compared with single stimulation with iron or TNF-alpha alone. Therefore, IL-24 was selected as an investigation target.

**Table 1:** Microarray analysis for control vs Fe or TNF-alpha stimulation on day 1.

	Fe	TNF-alpha	TNF-alpha + Fe
Homo sapiens colony stimulating factor 3 (granulocyte) (CSF3), transcript variant 1	48.280	25.387	815.427
Homo sapiens chemokine (C-X-C motif) ligand 2 (CXCL2)	8.827	4.844	22.277
Homo sapiens interleukin 24 (IL-24), transcript variant 3	8.410	10.925	86.005
Homo sapiens chemokine (C-X-C motif) ligand 5 (CXCL5)	7.394	9.362	26.913
Homo sapiens colony stimulating factor 3 (granulocyte) (CSF3), transcript variant 1,	5.574	2.942	96.450
Homo sapiens interleukin 24 (IL-24), transcript variant 3	4.465	3.164	45.411
Homo sapiens proline rich 18 (PRR18)	−2.685	1.334	−23.292
Homo sapiens olfactory receptor, family 4, subfamily E, member 2 (OR4E2)	−14.429	−13.842	−13.619
Homo sapiens H19, imprinted maternally transcript (non-protein coding) (H19)	−3.592	−7.407	−10.707

### IL-24 expression after iron stimulation

**Figure 3:**
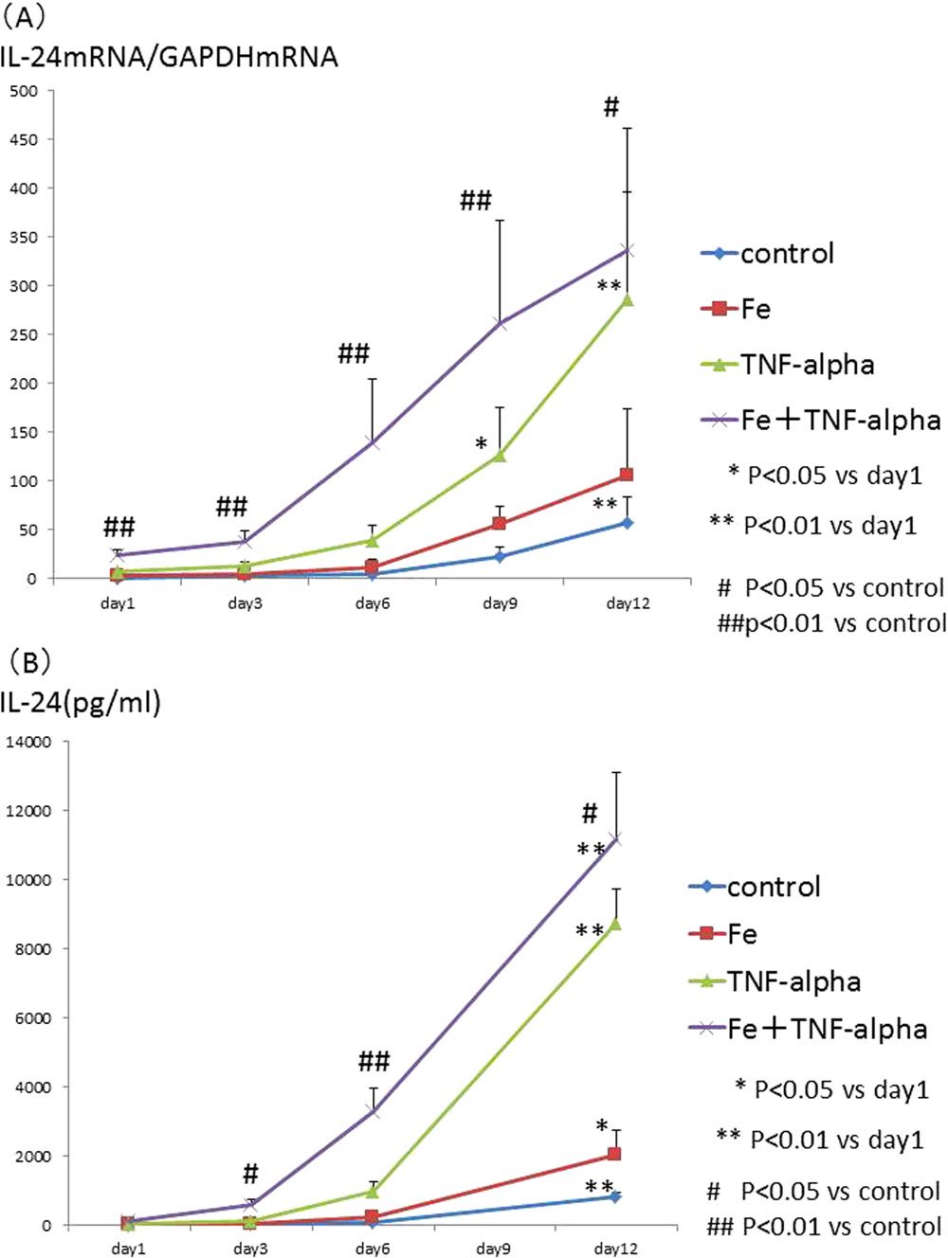
(**A**) Time course of IL-24 mRNA expression levels induced by iron, TNF-alpha or both iron and TNF-alpha stimulation. The time course of IL-24 gene expression was evaluated by real-time PCR on days 1, 3, 6, 9, and 12 after the addition of 100 µg/mL iron (holo-transferrin) and/or 1 ng/mL TNF-alpha to the calcification medium. The gene IL-24 expression level was enhanced by iron and TNF-alpha stimulation, and the increased gene expression level was maintained during the cell culture period. These experiments used one cell lines of HASMCs. (**B**) Time course of IL-24 by ELISA induced by iron, TNF-alpha or both iron and TNF-alpha stimulation. The time course of IL-24 protein expression was evaluated by real-time PCR on days 1, 3, 6, and 12 after the addition of 100 µg/mL iron (holo-transferrin) and/or 1 ng/mL TNF-alpha to the calcification medium. The IL-24 protein levels were enhanced by iron and TNF-alpha stimulation, and the increased gene expression level was maintained during the cell culture period. These experiments used one cell lines of HASMCs.

The time course of IL-24 gene expression was evaluated by real-time PCR on days 1, 3, 6, 9, and 12. The IL-24 gene expression levels were enhanced by iron and TNF-alpha stimulation, and the gene expression levels continued to increase during the calcification process (Fig. 3A). The IL-24 protein levels were also enhanced by iron and TNF-alpha stimulation (Fig. 3B), in parallel with the gene expression levels.

### Confirmation of the effect of IL-24 on calcification

**Figure 4:**
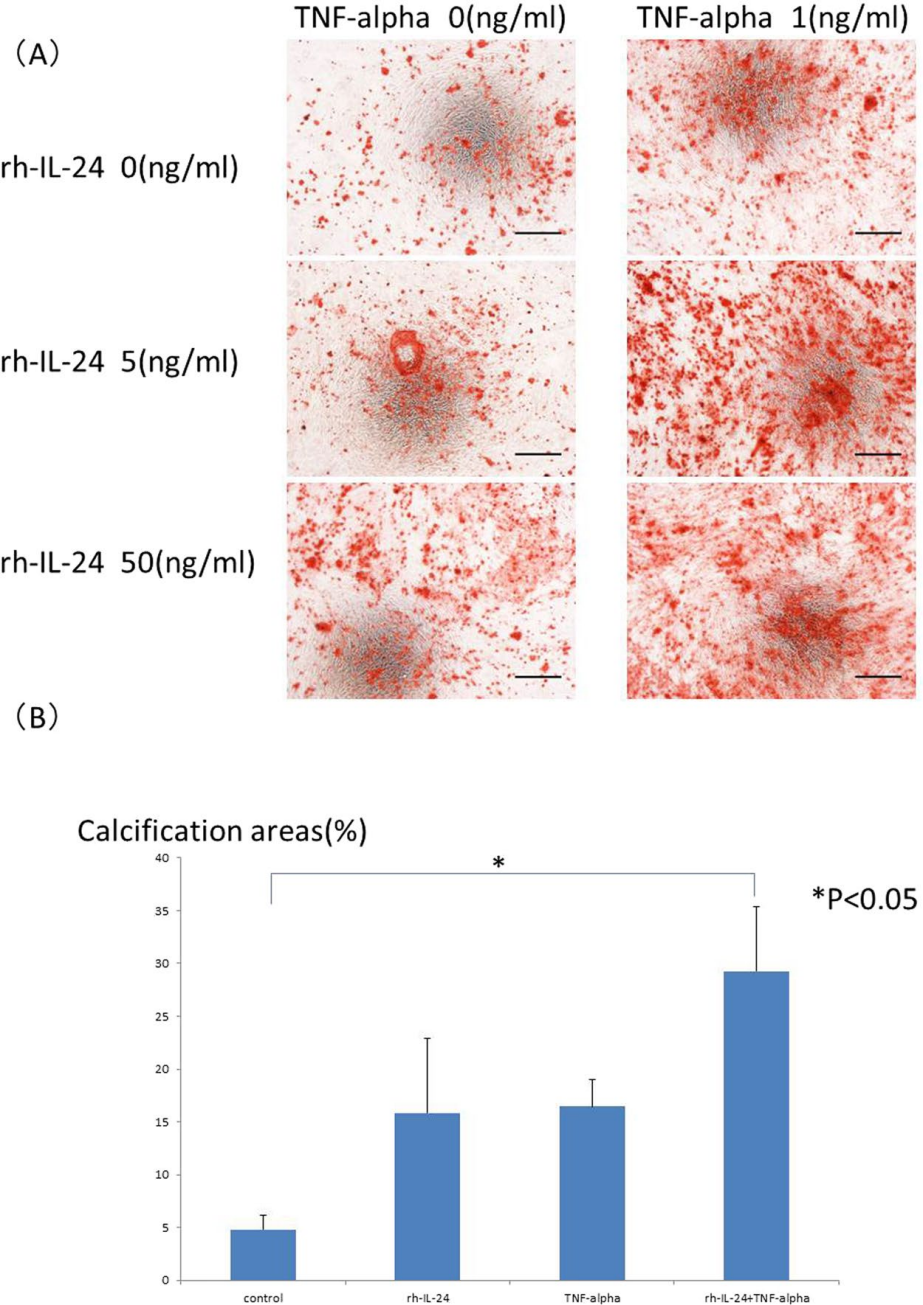
(**A**) Typical images of calcification of HASMCs induced by recombinant IL-24, TNF-alpha or both recombinant IL-24 and TNF-alpha. HASMCs were incubated with the calcification medium for 15–21 days in the presence of recombinant human IL-24 (0, 5, or 50 ng/mL) and TNF-alpha (0, or 1 ng/mL). Mineralized nodules of these cells were stained with Alizarin red, and typical images of calcification in HASMCs are shown. The addition of IL-24 increased calcification in these cells. TNF-alpha further enhanced the calcification. Black bars indicate 500 micrometers. (**B**) Quantification of calcification of HASMCs induced by recombinant IL-24 or TNF-alpha or both recombinant IL-24 and TNF-alpha by ImageJ software. The calcification areas of HASMCs stained with Alizarin red were quantified using ImageJ software. Recombinant IL-24 induced HASMC calcification, similar to iron-induced HASMC calcification. The 5 ng/mL dose of recombinant IL-24 and 1 ng/mL of TNF-alpha synergistically induced HASMC calcification. These experiments used two cell lines of HASMCs.

**Figure 5:**
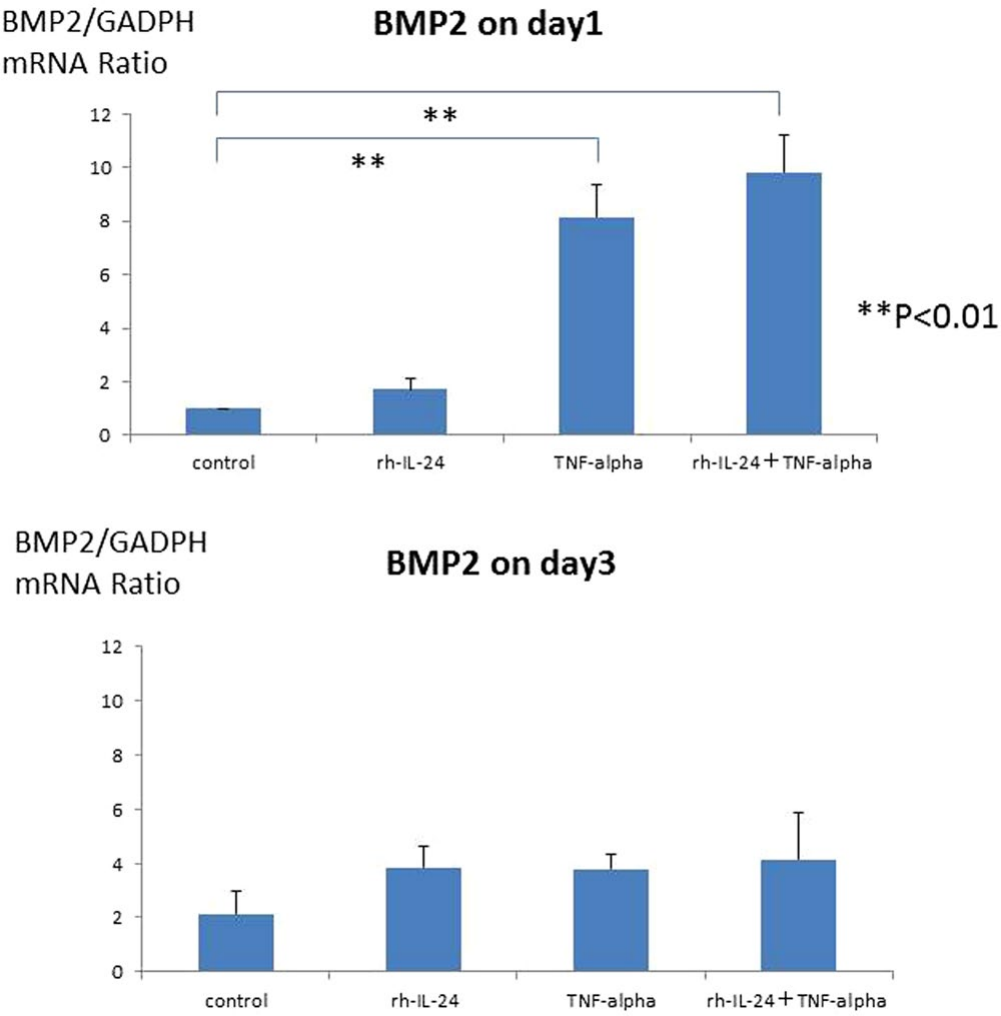
BMP2 expression was evaluated by real-time quantitative PCR following recombinant IL-24 or TNF-alpha stimulation or both recombinant IL-24 and TNF-alpha. To confirm the calcification pathway, BMP2 expression was evaluated by real-time quantitative PCR. BMP2 expression was significantly elevated by recombinant IL-24 and/or TNF-alpha stimulation on day 1 (P < 0.01), but BMP2 elevation was not observed on day 3. These experiments used one cell lines of HASMCs.

**Figure 6:**
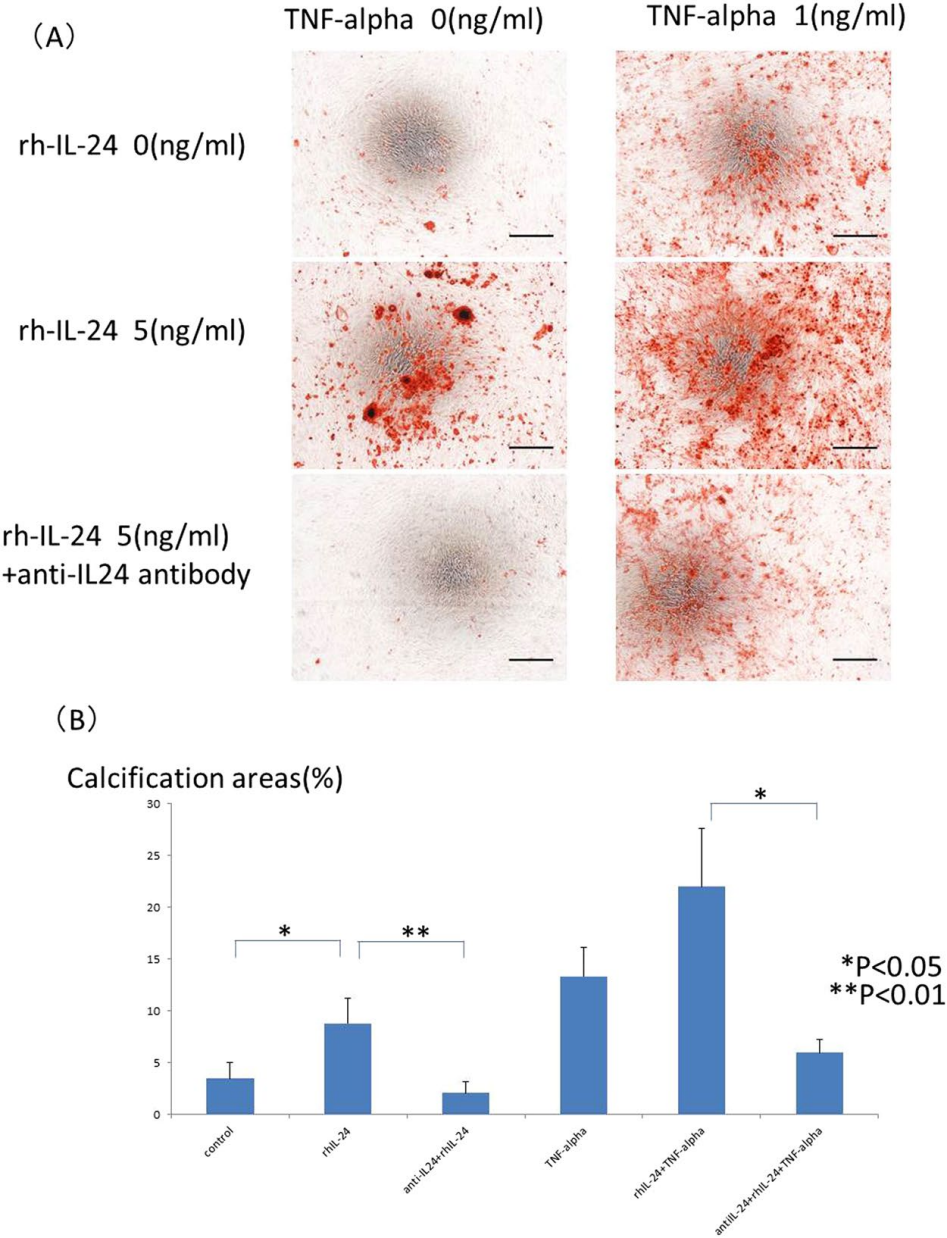
(**A**) Typical images of calcification of HASMCs induced by IL-24 and inhibition of calcification by anti-IL-24 antibody. To confirm the effect of IL-24 on HASMC calcification, the cells were incubated with the calcification medium for 15–21 days in the presence of recombinant human IL-24 (0 or 5 ng/mL) and TNF-alpha stimulation (0 or 1 ng/mL) with or without anti-IL24 antibody (0.5 μg/ml). Mineralized nodules of cells were stained with Alizarin red, and typical calcification images in HASMCs are shown. The anti-IL-24 antibody inhibited the calcification induced by IL-24 stimulation. Black bars indicate 500 micrometers. (**B**) Quantification of calcification of HASMCs induced by recombinant IL-24 or TNF-alpha and inhibition of the calcification by anti-IL-24 antibody by ImageJ software. The calcification areas of HASMCs stained with Alizarin red were quantified by ImageJ software. Recombinant IL-24 significantly induced HASMC calcification (P < 0.05), and the anti-IL-24 antibody significantly inhibited the calcification (P < 0.01). The calcification induced by both recombinant IL-24 and TNF-alpha was also significantly inhibited by the anti-IL-24 antibody (P < 0.05). These experiments used two cell lines of HASMCs.

If iron induced calcification independent of IL-24, IL-24 might not induce calcification. If iron induced calcification through IL-24, IL-24 should induce calcification. To confirm which possibility was correct, recombinant IL-24 was added to the calcification medium in place of iron with or without TNF-alpha (1 ng/mL). Calcification was enhanced by recombinant IL-24 stimulation in a dose-dependent manner, similar to iron stimulation. Typical calcification images in HASMCs are shown in Fig. 4A. The calcification areas were quantified by ImageJ software, and the quantification results are shown in Fig. 4B. IL-24 induced HASMCs calcification. TNF-alpha also induced HASMCs calcification in a dose-dependent manner. Interestingly, either 5 or 50 ng/mL IL-24 and 1 ng/mL TNF-alpha synergistically induced HASMCs calcification (Fig. 4A). These results were quantified by ImageJ software, and the significant increase in calcification induced by IL-24 (50 ng/mL) and TNF-alpha was confirmed (Fig. 4B). To confirm the calcification pathway, BMP2 expression was evaluated by real-time quantitative PCR. BMP2 was significantly elevated by TNF-alpha stimulation or by both IL-24 (50 ng/mL) and TNF-alpha stimulation (Fig. 5) on day one, similar to our observation with the iron stimulation conditions. However, there was no significant change in BMP2 expression under either condition on day 3 (Fig. 5). To clarify the role of IL-24 in the calcification induced by iron, we added a sufficient amount of an anti-IL-24 antibody (0.5 μg/mL) to cancel the 5 ng/mL IL-24 added to the medium and the IL-24 stimulation. The anti-IL-24 antibody reversed the effect of IL-24. Typical calcification images in HASMCs are shown in Fig. 6A, and the quantification of the calcification area is shown in Fig. 6B. Because the increase in IL-24 induced by iron stimulation was too large to be reversed by the anti-IL-24 antibody, the direct inhibition of iron stimulation by the anti-IL-24 antibody could not be assessed.

## Discussion

Human aortic vascular smooth muscle cells (HASMCs), in which Moenckeberg’s arteriosclerosis is induced in CKD patients, demonstrated calcification induced by iron and pro-inflammatory cytokine stimulation (TNF-alpha). The calcification of HASMCs was synergistically enhanced by both iron and TNF-alpha stimulation. In the early phase of calcification, microarray analysis revealed up-regulation of IL-24, which was confirmed at the mRNA level by real-time PCR and at the protein level by ELISA. The stimulation of HASMCs by IL-24, instead of iron-induced calcification, indicated that the calcification induced by iron stimulation might be mediated by IL-24. The anti-IL-24 antibody reversed the effect of IL-24, supporting the important role of IL-24 in HASMCs calcification.

An increase in BMP2 was demonstrated as a major step in osteochondrocytic differentiation during the calcification process^16–18^. The increase in BMP2 in our results indicated that iron stimulation might have induced calcification in vascular smooth muscle cells through the BMP2 pathway. Moreover, activation of known calcification markers supported this pathway rather than calcification related to the cell death process 19–21. Elevation of Runx2 and human OPG might be caused by the calcification medium itself because there were no changes under stimulation conditions or under control conditions. The time courses of RANKL were similar to those of calcification under iron and/or TNF-alpha stimulation, which might account for the enhancement of calcification induced by TNF-alpha and iron stimulation. The increase of activity of alkaline phosphate and BMP2 which return to the basal levels during calcification periods might indicate the possibility that HASMCs might trans-differentiated into mineralizing cells^19^. Holo-transferrin (holo-Tf) was used for iron stimulation; therefore, this type of iron was absorbed into the cultured vascular smooth muscle cells through transferrin receptor 1^10,20^, and the iron in the cells might have induced oxidative stress through the Fenton reaction^6^. Although several reports have indicated that iron enhances calcification in endothelial cells^11,12^, reports concerning the relationship between iron and vascular smooth muscle cells have been limited. Iron overload in vascular smooth muscle cells with inflammatory stimulation might account for the mechanism of vascular calcification (Moenckeberg’s arteriosclerosis) in vascular media cells, which is usually observed in CKD patients.

IL-24 was identified as melanoma differentiation-associated gene 7 (mda-7)^21^. IL-24 includes an IL-10 signature and consists of 7 exons and 6 introns on chromosome 1q^22^, which contains a cluster of genes that encode several members of the IL-10 family of cytokines^23^. The combination of the IL-10 family signature, genomic location and shared receptors led to the cytokine being renamed IL-24^24^. IL-24 has been evaluated as an anti-cancer molecule because cancer cells transfected with IL-24 exhibit inhibited cell growth and colony formation. This phenomenon has been observed in melanoma cells^21^ and many other cancer cells^22,25,26^. In one anti-cancer mechanism, IL-24 induces apoptosis through reactive oxygen species (ROS) generation^27^. This group also reported that ROS induction sensitized pancreatic cells to apoptosis induced by mda-7/IL-24^27^. The apoptosis-inducing property of IL-24 was partially due to ROS generation^28,29^. Moreover, IL-24 was reported to inhibit antioxidant responses^30^. Our study indicated that iron stimulation enhanced IL-24 gene expression and that IL-24 stimulation itself caused calcification in vascular smooth muscle cells. Iron stimulation of vascular smooth muscle cells might induce ROS stress through the Fenton reaction and increase IL-24 gene expression level, resulting in apoptosis. Apoptotic cells are considered the core of calcification^16,31–33^.

In terms of the relationship between IL-24 and calcification in vascular smooth muscle cells, one report seemed to indicate that IL-24 inhibited calcification of these cells^34^. However, this study used completely different procedures. First, IL-24 was added to the cultured cells only during the initial 12 hours, whereas we continued to add IL-24 to the medium to mimic the IL-24 elevation induced by iron stimulation. Second, the conditions for the evaluation of calcification were different between our study and the previous study. Culturing of human aortic smooth muscle cells in calcification medium basically results in calcification after sufficient cell culture periods. In the previous study, the control cells manifested calcification, while the control cells in our study did not show calcification because the cells were evaluated before all of the culture cells calcified. Third, the IL-24 concentration in the inhibition study was 50–70 ng/mL, whereas the concentration in this study was 5 ng/mL, which was sufficient to promote calcification. Fourth, we used 5 ng/mL IL-24 to confirm the inhibition of calcification by anti-IL-24 antibody, whereas the inhibition experiment used 50 ng/mL IL-24 with anti-IL-24 antibody, resulting in partial cancelation of IL-24 by the anti-IL-24 antibody. These different conditions might result in different results. In terms of role of IL-24 against reactive oxygen stress, it was reported IL-24 could play a protective role, although this study used endothelial cells where calcification did not occur^35^. Moreover, it was reported that the endothelium might be a source of osteoprogenitor cells in vascular calcification^36^. Our results might be altered by the presence of the endothelium cells, although the vascular smooth muscle cells without endothelium cells had been used in order to reveal the mechanism of vascular calcification. Important molecules sometimes have offensive and defensive role in the different cells or in the different situations. There was still some possibility that IL-24 have some protective role in our study, although IL-24 itself induced calcification in HASMCs. The role of IL-24 in vascular calcification should be confirmed using physiological model such as experimental animal model.

Iron overload had been reported to induce many complications, such as acceleration of arteriosclerosis^9,10^, infectious diseases^37,38^, cardiovascular events^38^, and poor prognosis^39^ in CKD patients. Our results might support recommendations to avoid iron overload in CKD patients in terms of vascular calcification. However, it is impossible to strictly prevent the effects of iron because iron is essential for humans. Therefore, IL-24 might be a new target for anti-calcification therapy, instead of iron itself.

In conclusion, iron-induced calcification in vascular smooth muscle cells occurred via IL-24. IL-24 was increased during the calcification process induced by iron, and IL-24 itself caused calcification in the absence of iron.

## Material and Methods

### Effects of iron and TNF-alpha on calcification in smooth muscle cells

Human aortic smooth muscle cells (HASMCs) (Lonza, Basel, Switzerland) were cultured with the “SmGM-2 SingleQuot Kit Suppl. & Growth Factors (Cat#CC3182, Lonza)” at 37 °C in a humidified atmosphere with 5% CO2. Briefly, this medium kit included 5% FBS, FGF-B, EGF, and insulin. Six cell lines of HASMCs were used, and the profiles of these cell lines are shown in Supplemental Table 1. All of six cell lines of HASMCs could induce calcification, but two of six cell lines of HASMCs could induce calcification by calcification medium itself, which suggesting that it was hard to confirm the effect of iron and/or TNF-alpha on calcification process. 4 cell lines could induce calcification and the enhancement of calcification induced by iron and/or TNF-alpha could be confirmed. We used 2 cell lines for following experiments. HASMCs were used in passages 6–7 for the experiments. To induce calcification, the HASMCs were switched at confluence to the calcification medium (DMEM, 15% FBS and Pi (final concentrations 3.5 mmol/L) with penicillin (100 U/mL) and streptomycin (100 mg/mL)). To confirm the effects of iron stimulation, holo-transferrin (holo-Tf) (SIGMA-ALDRICH, Tokyo, Japan) (0, 30, or 100 µg/mL) was added to the calcification medium. To confirm the effects of the inflammatory conditions, TNF-alpha (0, 1, or 10 ng/mL, PeproTech, Inc., Rocky Hill, NJ, USA) was also added to the medium. The medium was half-changed every 2–3 days for 15–21 days. During the cell culture period, the iron and TNF-alpha concentrations were maintained by the addition of these factors to the new medium. For the time-course experiments, the first day of culture in the calcification medium was defined as day 0. Alizarin red staining was used to evaluate the calcification. Briefly, HASMCs in culture plates were washed with PBS and fixed in cold 70% ethanol for 1 hour at 4 °C. Then, the HASMCs were washed in distilled water and exposed to fresh 2% Alizarin red (Sigma-Aldrich, St. Louis, MO, USA) for 5 minutes. After staining, the cells were washed with distilled water at least 5 times, washed once in PBS for 15 minutes, and then photographed under a microscope (TE300-HM-2, Nikon, Tokyo, Japan) equipped with a charge-coupled device (CCD) digital camera (Nikon) and software. The photographs were imaged with a scanner (GT-X970, EPSON, Tokyo, Japan). The images of the nodules were quantified using image analysis software (ImageJ, National Institutes of Health, Bethesda, MD, USA) to determine the area of the mineralized nodules. To confirm the calcification pathway, BMP2 expression was evaluated by quantitative real-time PCR on days one and three. Moreover, BMP2 expression was evaluated from day 1 to day 12, during the calcification process.

To confirm the calcification process, known calcification markers, such as Runx2, MSX2, RANKL, and human osteoprotegrin (hOPG), were evaluated by quantitative real-time PCR during the calcification process. The PCR primers used in these experiments are shown in Supplemental Table 2. Alkali phosphatase activities were also evaluated by enzymatic activity/protein concentrations using Alkaline Phosphatase Assay (SenoLyte pNPP Alkaline Phosphatase Assay Kit, ANA SPEC, Fremont, CA, USA). Protein concentrations were evaluated by BCA protein assay (Pierce BCA protein assay Kit, Thermo Fisher Scientific, MA, USA). Alkali phosphatase activities/protein concentrations were used for analyses.

### Microarray analysis of the early response to iron stimulation

After confirmation of the effects of calcification induced by iron and TNF-alpha stimulation, we performed microarray analysis using mRNA on day one to reveal the early gene response to iron or TNF-alpha stimulation, compared to usual calcification in HASMCs without TNF-alpha and iron stimulation. The microarray methods were described elsewhere. Briefly, the quality of the RNA samples was examined using the RNA 6000 Nano LabChip Kit (p/n 5065–4476) on the Agilent 2100 Bioanalyzer platform (G2940BA, Agilent Technologies, Inc., Palo Alto, CA, USA). Total RNA (500 ng) from HASMCs (on day one with: 1) holo-Tf (100 µg/mL) only; 2) TNF-alpha (1 ng/mL) only; 3) both holo-Tf (100 µg/mL) and TNF-alpha (1 ng/mL); and 4) without both holo-Tf and TNF-alpha (control)) was reverse-transcribed using oligo-dT primers containing the T7 RNA polymerase promoter sequence. The complementary DNAs (cDNAs) were subjected to *in vitro* transcription using the T7 RNA polymerase to label them with Cy3 or Cy5 (CyDye, GE Healthcare, Biosciences Corp., Piscataway, NJ, USA). The Cy-labeled cRNA from the HASMCs was mixed with the same amount of reverse color Cy-labeled product from an equal amount of cRNA from control HASMCs. The labeled cRNAs were fragmented to an average size of approximately 50–100 nt by heating at 60 °C in the presence of 10 mM ZnCl_2_. Then, the samples were added to hybridization buffer containing 1 M NaCl, 0.5% sodium sarcosine, 50 mM MES (pH 6.5) and formamide to a final concentration of 30% in a final volume of 3 mL. Hybridization with Agilent’s whole human genome microarray (Hu44K) (Agilent Technologies, Inc. Santa Clara, CA, US) was conducted at 40 °C. The microarray sequences were selected from RefSeq (a collection of non-redundant mRNA sequences; http://www.ncbi.nlm.nih.gov/gquery.fcgi?term=RefSeq) and from expressed sequence tag (EST) contigs (http://www.phrap.org/green_group/est_assembly/human/gene_number_methods.html). Each mRNA or EST contig was represented on the Hu44K microarray by a single 60 mer oligonucleotide, chosen by the oligonucleotide probe design program. After hybridization, the slides were washed and scanned using an Agilent confocal laser scanner (G2565BA). The fluorescence intensities of the scanned images were quantified, corrected for background noise and normalized. Fluorophore reversal (dye swap) duplicates were used in the two-color DNA microarray experiments.

### IL-24 expression after iron stimulation

IL-24 and CSF3 were selected from the results of microarray analysis as analysis targets because they were increased by iron stimulation and TNF-alpha stimulation. Moreover, both iron and TNF-alpha stimulation synergistically increased IL-24 and CSF3 gene expression in HASMCs. However, this synergistic increase in CSF3 gene expression could not be confirmed (data not shown); therefore, gene expression of IL-24 was investigated during the calcification process period. The IL-24 expression levels after iron and TNF-alpha stimulation were evaluated by real-time quantitative PCR on days 1, 3, 6, 9, and 12. Total RNA was isolated from HASMCs with the TRIzol Reagent (Thermo Fisher, Waltham, MA, USA), according to the manufacturer’s protocol. To obtain total RNA from TNF-alpha- and iron-stimulated cells, we treated confluent HASMCs with or without TNF-alpha (1 ng/mL) and iron (100 µg/mL), followed by the calcification medium. We obtained cDNA from the total RNA using the High Capacity RNA-to-cDNA Kit (Invitrogen, Carlsbad, CA, USA). The primer pairs used in this study are provided in Table 2. cDNA was relatively quantified by real-time PCR using the SYBR Green PCR Master Mix (Invitrogen) in a real-time PCR machine (7500–01, Applied Biosystems, Foster City, CA, USA). The results are expressed as the ratio of the target PCR product relative to the GAPDH product. IL-24 protein levels after iron and TNF-alpha stimulation were evaluated by enzyme-linked immunosorbent assay (ELISA). The IL-24 protein concentrations in the supernatants were measured using the OmniKine™ Human IL-24 ELISA Kit (Assay Biotechnology Company, Sunnyvale, CA, USA), in accordance with the manufacturer’s protocols.

**Table 2:** Nucleotide sequence of each primer for PCr.

Genes		Primer Sequence
IL-24	Forward	5′-GCTGCAGCAGGAGGTTCT-3′
	Reverse	5′-GCAGGGTGTGGACAAGGTAA-3′
GAPDH	Forward	5′-GCACCGTCAAGGCTGAGAAC-3′
	Reverse	5′-ATGGTGGTGAAGACGCCAGT-3′
BMP2	Forward	5′-CCAGCTTCTCCTTTCTCCCT-3′
	Reverse	5′-CCATGGTCGACCTTTAGGAG-3′

### Confirmation of the effect of IL-24 on calcification

There were two possibilities: that iron might induce calcification through IL-24 or that iron might induce calcification and increase IL-24 independently. To confirm that IL-24 induced calcification instead of iron or not, recombinant IL-24 (R&D Systems, Minneapolis, MN, USA) (0, 5, or 50 ng/mL) was added to the calcification medium instead of iron in combination with TNF-alpha (0 or 1 ng/mL). The concentrations of recombinant IL-24 and TNF-alpha were maintained by adding these cytokines to the calcification medium whenever the medium was changed. Calcification was evaluated using Alizarin red staining. To confirm the calcification pathway, BMP2 expression was evaluated on days one and three by quantitative real-time PCR. To clarify the effect of IL-24, a human IL-24 antibody (0.5 μg/ml) (R&D Systems, Minneapolis, MN, USA) was added to the calcification medium with the IL-24 (5ng/ml).

### Statistical analysis

The statistical analyses were performed with the EZR software program (Saitama Medical Centre, Jichi Medical University, Tokyo, Japan). The data were assessed for significant differences using Bonferroni’s multiple comparison test. A P value of 0.05 was considered statistically significant (*P < 0.05, **P < 0.01).

## Electronic supplementary material

Supplementary Information
