# Supplementary material for: Iron-induced calcification in human aortic vascular smooth muscle cells through interleukin-24 (IL-24), with/without TNF-alpha
Source: Sci Rep. 2018 Jan 12;8:658. doi: 10.1038/s41598-017-19092-1 (PMC5766506; doi:10.1038/s41598-017-19092-1)
Supplement: Supplementary file 1 — Supplementary Information [file 41598_2017_19092_MOESM1_ESM.pdf]

Iron-induced calcification in human aortic vascular smooth muscle cells through interleukin-24 (IL-24) with/without TNF-alpha

Sayuri Kawada, Yasuyuki Nagasawa, Mutsuki Kawabe, Hideki Ohyama,
Aritoshi Kida, Nahoko Kato-Kogoe, Masayoshi Nanami, Yukiko Hasuike,
Takahiro Kuragano, Hiromitsu Kishimoto, Keiji Nakasho, Takeshi Nakanishi

Supplemental Fig 1

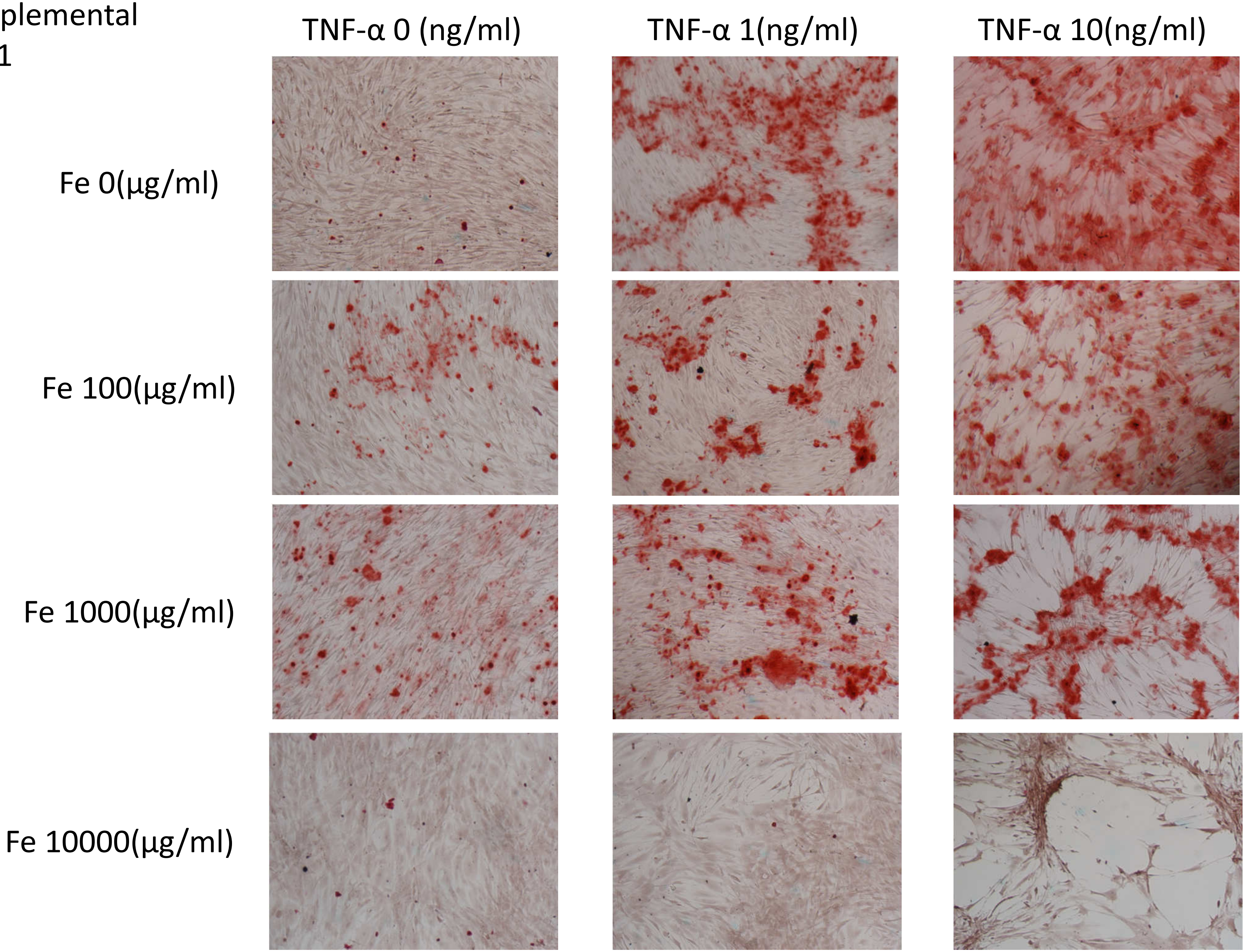

Supplemental
Fig 2

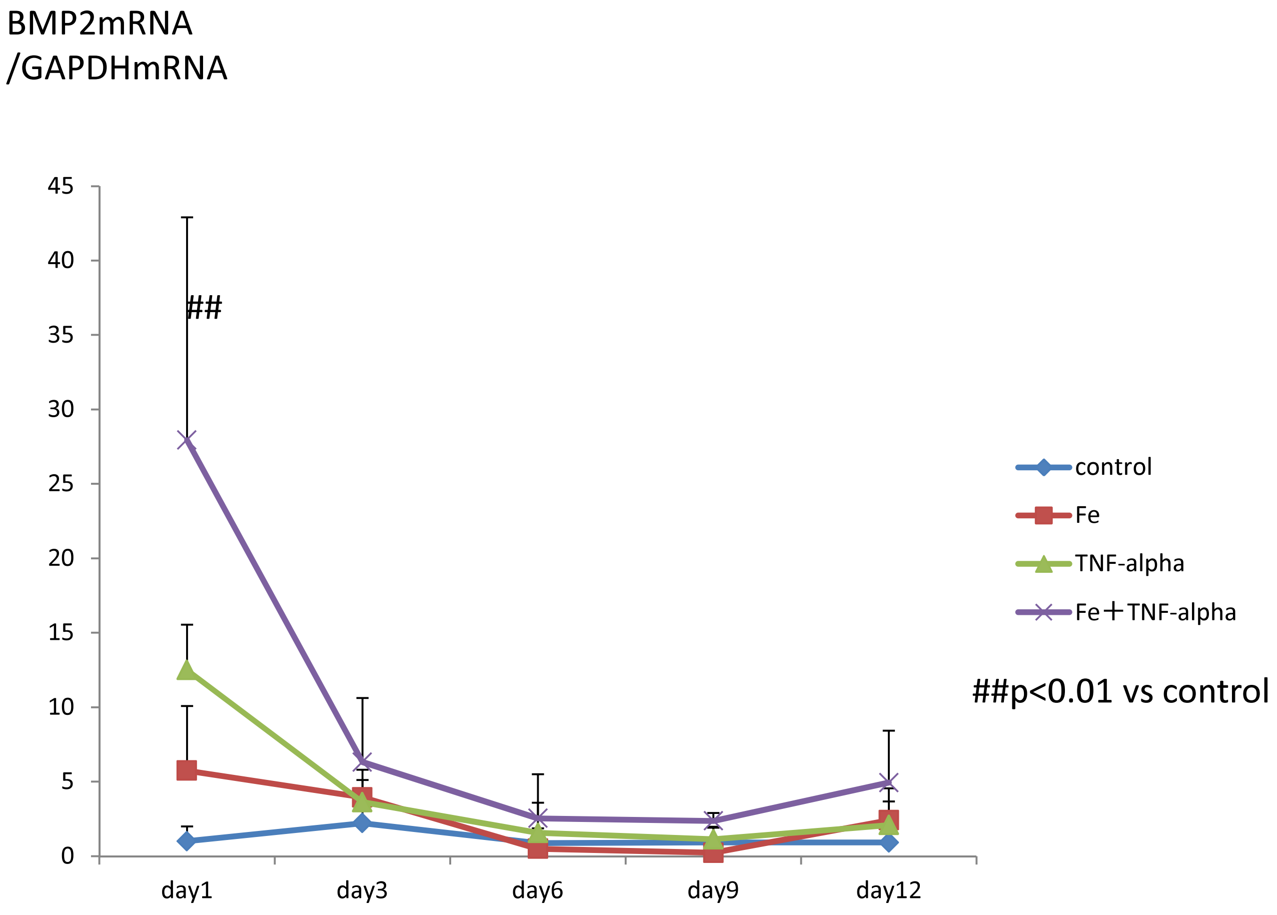

Supplemental
Fig 3

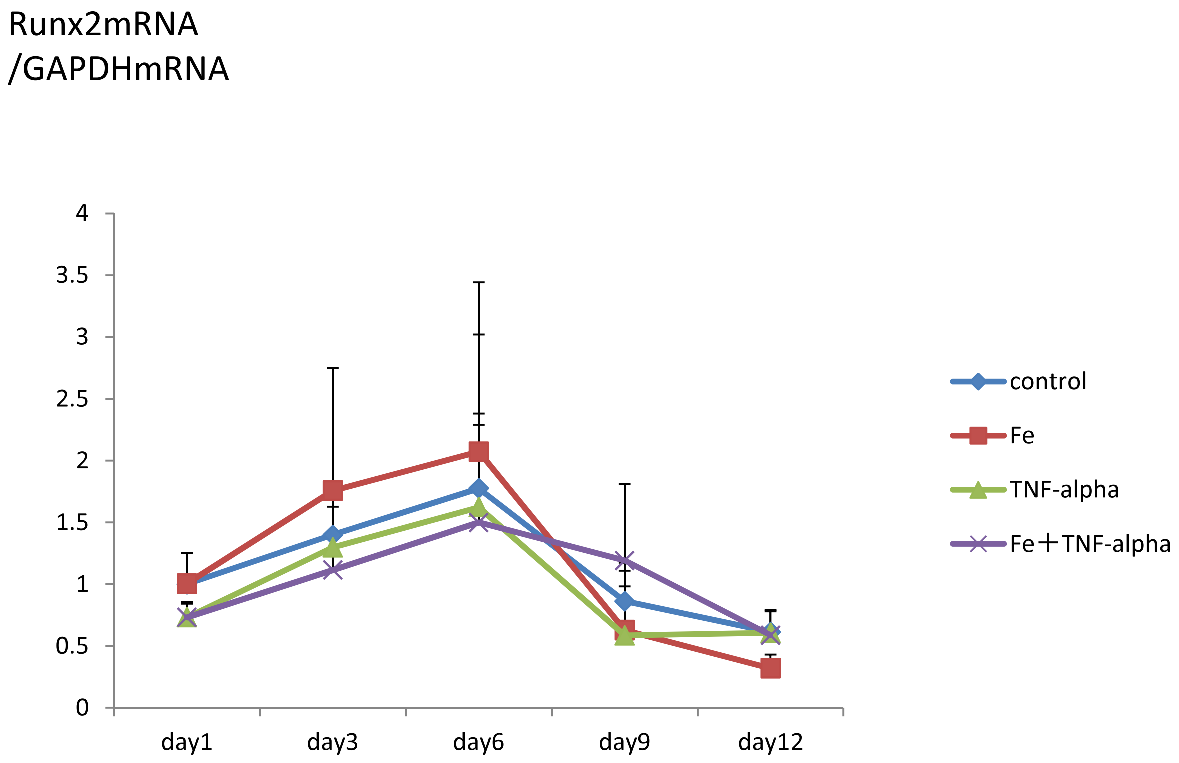

Supplemental
Fig 4

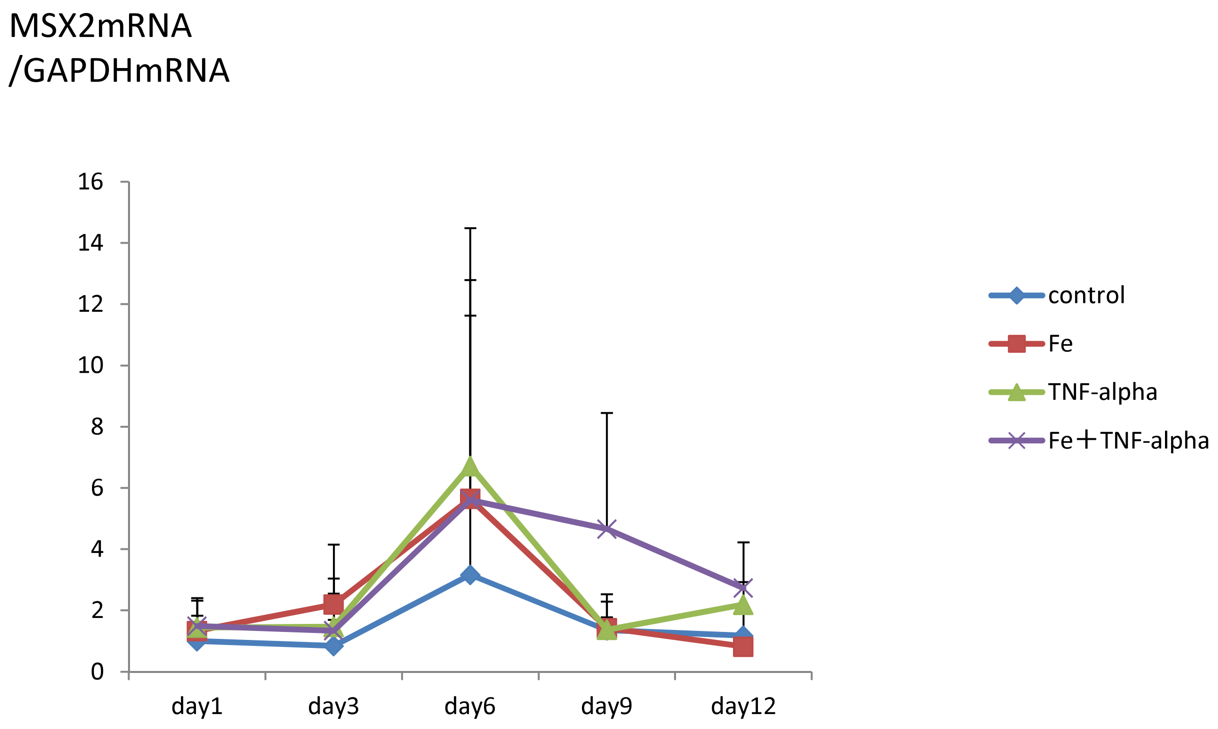

Supplemental
Fig 5

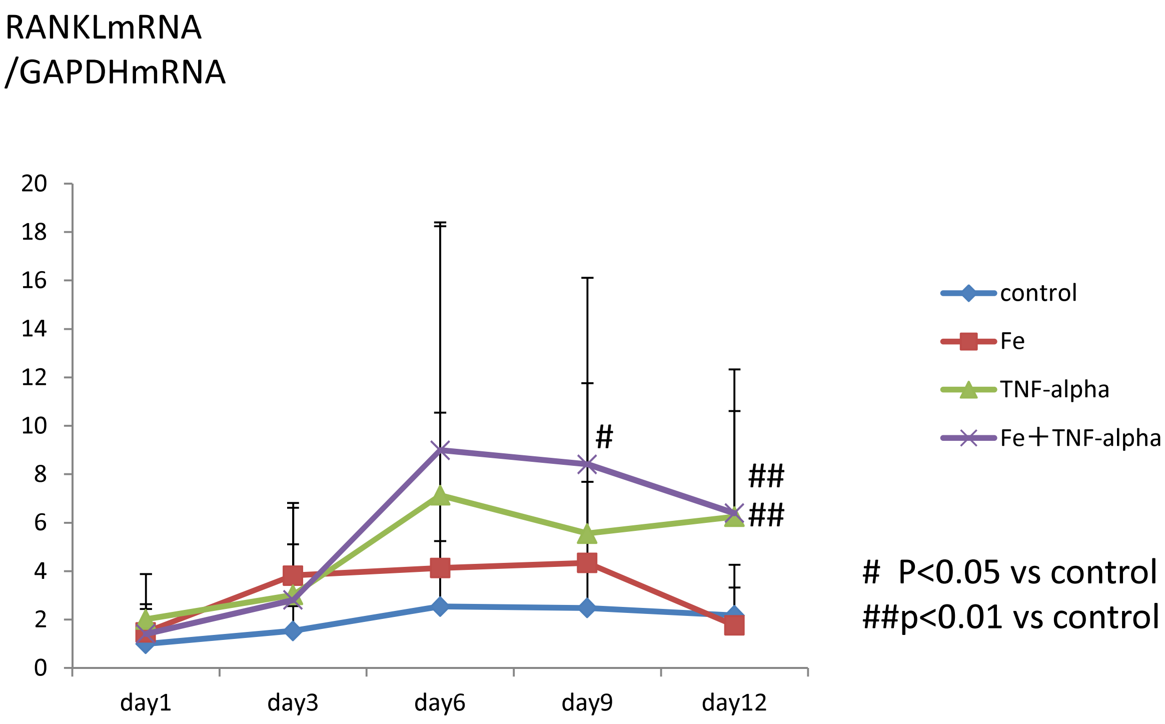

Supplemental
Fig 6

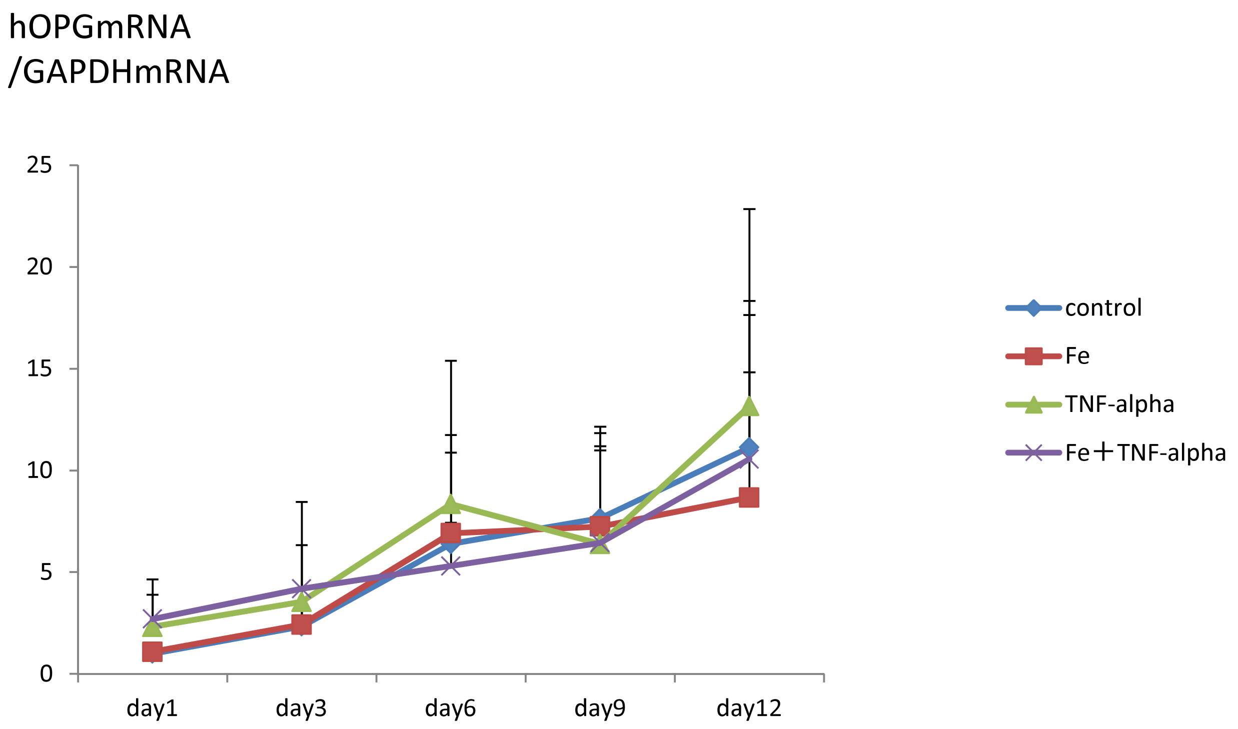

Supplemental
Fig 7

ALP activity/Protein

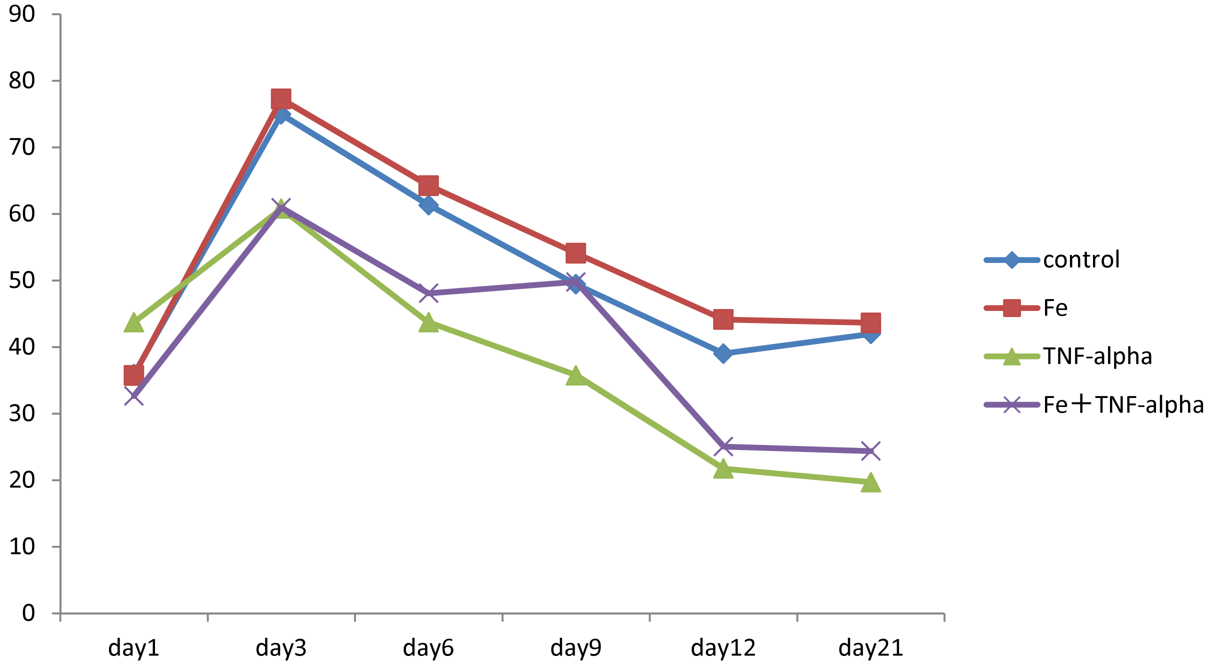

Supplemental table 1. Backgrounds of human aortic smooth muscle cells used in this study.

Lot Number	Age	Sex	Ethnic
7F4356	21	M	Caucasian
7F4322	20	M	Hispanic
289735	57	F	Black
289196	81	M	Caucasian
305413	27	M	Orient
335663	22	M	Caucasian

Supplemental table 2. PCR primers for confirmation of calcification process

Runx2
F : CCG GAG TGG ACG AGG CAA GAG TT
R : AGC TTC TGT CTG TGC CTT CTG GG

hOPG
F : GGC AAC ACA GCT CAC AAG AA
R : CTG GGT TTG CAT GCC TTT AT

Msx2
F : GAT TTG GGT GGG GGT TTA GT
R : TGA CAA TCC CAT TTT CAC CA

RANKL
F : ATC ACA GCA CAT CAG AGC AGA G
R : GGA CAG ACT CAC TTT ATG GGA ACC

Supplemental Figure Legends

Supplemental Figure 1 Typical images of calcification and cell death in human aorta vascular smooth muscle cells induced by high concentrations of iron, TNF-alpha or both iron and TNF-alpha.

To confirm the safety of iron in human aortic smooth muscle cells (HASMCs), the cells were cultured with the calcification medium for 15-21 days, supplemented with holo-transferrin (holo-Tf) (0, 100, 1000 or 10000 μg/mL) and TNF-alpha (0, 1, or 10 ng/mL). Mineralized cell nodules were stained with Alizarin red, and typical calcification images of HASMCs are shown. The high concentration (10000 μg/mL) stimulation induced cell death and suppressed calcification.

Supplemental Figure 2 Time course of BMP2 mRNA expression levels following iron, TNF-alpha or both iron and TNF-alpha stimulation.

The time course of IL-24 gene expression was evaluated by real-time PCR on days 1, 3, 6, 9, and 12 after the addition of 100 μg/mL of iron (holo-transferrin) and/or 1 ng/mL of TNF-alpha to the calcification medium. The gene BMP2 expression level was enhanced by iron and TNF-alpha stimulation at day 1, and the BMP2 gene expression level decreased to basal levels after day 3. These experiments used one cell lines of HASMCs.

Supplemental Figure 3 Time course of Runx2 mRNA expression levels following iron, TNF-alpha or both iron and TNF-alpha stimulation.
The time course of Runx2 gene expression was evaluated by real-time PCR on days 1, 3, 6, 9, and 12 after the addition of 100 µg/mL iron (holo-transferrin) and/or 1 ng/mL TNF-alpha to the calcification medium. The gene expression level of Runx2 seemed to be enhanced by iron and/or TNF-alpha stimulation at day 6, and the Runx2 gene expression level decreased to basal levels after day 9. These experiments used one cell lines of HASMCs.

Supplemental Figure 4 Time course of MSX2 mRNA expression levels following iron, TNF-alpha or both iron and TNF-alpha stimulation.
The time course of MSX2 gene expression was evaluated by real-time PCR on days 1, 3, 6, 9, and 12 after the addition of 100 µg/mL iron (holo-transferrin) and/or 1 ng/mL TNF-alpha to the calcification medium. The gene expression level of MSX2 seemed to be enhanced by iron and/or TNF-alpha stimulation on day 6 without statistical significance, and the MSX2 gene expression level was seemed return to the basal level after day 9. These experiments used one cell lines of HASMCs.

Supplemental Figure 5 Time course of RANKL mRNA expression levels following iron, TNF-alpha or both Iron and TNF-alpha stimulation.
The time course of RANKL gene expression was evaluated by real-time PCR on days 1, 3, 6, 9, and 12 after the addition of 100 μg/mL iron (holo-transferrin) and/or 1 ng/mL TNF-alpha to the calcification medium. The gene expression level of RANKL was significantly enhanced by iron and/or TNF-alpha stimulation after day 9, and the RANKL gene expression level was enhanced by TNF-alpha stimulation on day 12. These experiments used one cell lines of HASMCs.

Supplemental Figure 6 Time course of human osteoprotegrin (hOPG) mRNA expression levels following iron, TNF-alpha or both iron and TNF-alpha stimulation.
The time course of hOPG gene expression was evaluated by real-time PCR on days 1, 3, 6, 9, and 12 after the addition of 100 μg/mL iron (holo-transferrin) and/or 1 ng/mL TNF-alpha to the calcification medium. The gene expression level of hOPG seemed to increase gradually without statistical significance. These experiments used one cell lines of HASMCs.

Supplemental Figure 7 Time course of alkaline phosphatase activity/protein following iron, TNF-alpha or both iron and TNF-alpha stimulation.
The time course of alkaline phosphatase activity/protein was evaluated by enzymatic activity/protein on days 1, 3, 6, 9, 12 and 21 after the addition of 100 μg/mL iron (holo-transferrin) and/or 1 ng/mL TNF-alpha to the calcification medium. The alkaline phosphatase activity/protein l seemed to increase on day 3 and return to the basal level without statistical significance. These experiments used one cell lines of HASMCs.
